# Analysis of High-Throughput Flow Cytometry Data Using plateCore

**DOI:** 10.1155/2009/356141

**Published:** 2009-10-11

**Authors:** Errol Strain, Florian Hahne, Ryan R. Brinkman, Perry Haaland

**Affiliations:** ^1^FDA-Center for Food Safety and Nutrition, HFS-013 5100 Paint Branch Parkway, College Park, MD 20740, USA; ^2^Fred Hutchison Cancer Research Center, M2-B514 P.O. Box 19024 Seattle, WA 98109, USA; ^3^Terry Fox Laboratory, British Columbia Cancer Agency, 675 West 10th Avenue, Vancouver, BC, Canada V5Z 1L3; ^4^BD Technologies, 21 Davis Dr, Research Triangle Park, NC 27709, USA

## Abstract

Flow cytometry (FCM) software packages from R/Bioconductor, such as flowCore and flowViz, serve as an open platform for development of new analysis tools and methods. We created plateCore, a new package that extends the functionality in these core packages to enable automated negative control-based gating and make the processing and analysis of plate-based data sets from high-throughput FCM screening experiments easier. plateCore was used to analyze data from a BD FACS CAP screening experiment where five Peripheral Blood Mononucleocyte Cell (PBMC) samples were assayed for 189 different human cell surface markers. This same data set was also manually analyzed by a cytometry expert using the FlowJo data analysis software package (TreeStar, USA). We show that the expression values for markers characterized using the automated approach in plateCore are in good agreement with those from FlowJo, and that using plateCore allows for more reproducible analyses of FCM screening data.

## 1. Introduction

While there are a number of different software packages available for analysis of FCM data, these programs are often ill-suited to the development of new methods needed for analyzing high-throughput FCM studies. Flow Cytometry-High-Content Screening (FC-HCS) experiments generate large volumes of data [1, 2], which requires a systematic approach to preprocessing, gating (i.e., filtering), and summarizing results for robust analyses. Current FC-HCS data analysis methods often use a combination of software packages for different parts of the analysis. The raw FCM files are processed and gated using FCM specific software, such as FlowJo or FCS Express (De Novo Software, USA). Results are then exported, and statistical analysis is performed in packages like MATLAB (USA) and R (http://www.r-project.org/) [3]. Unfortunately, this approach to FC-HCS analysis results in methods that are semiautomated at best, and they often require significant subjective and error-prone manual intervention to identify cells of interest [4]. It is therefore desirable to develop programmatic approaches to process FCM data so that FC-HCS analysis pipelines are robust, objective, and able to match the high-throughput capacity of modern cytometers.

FCM packages available through the Bioconductor [3] project provide an open platform that can be used by cytometrists, bioinformaticians, and statisticians to collaboratively develop new methods for automated FC-HCS analysis. The basic data processing tools for importing, transforming, gating, and organizing raw FCM data are in the flowCore package [5] and the visualization functions are in flowViz [6]. The Bioconductor model for FCM data analysis facilitates the development of new analysis methods, since the overhead associated with accessing and visualizing FCM data is handled by flowCore and flowViz. The availability of flowCore and flowViz has enabled the creation of new tools for quality assessment of large FCM experiments, such as flowQ [7], and for model-based clustering and automated gating, such as flowClust [8].

We have developed an R package (plateCore) that also takes advantage of the functionality in flowCore and flowViz to create methods and data structures for processing large, plate-based FCM data sets. Additionally, we have implemented new tools to make it easier to integrate textual descriptions of plate layouts and also to perform automated gating based on nonparametric analysis of negative control wells. This study presents results from an automated plateCore analysis of a PBMC lymphocyte BD FACS CAP (Combinational Antibody Profile) data set, which included 189 different antibody-dye conjugates and their controls arranged on 5 replicate 96-well plates. The output of plateCore was compared to an analysis by an expert cytometrist using FlowJo, one of the standard FCM analysis programs, to evaluate the performance of the automated approach.

plateCore is not designed to be a graphical user interface driven tool, but rather to help develop a standardized platform for the analysis of FC-HCS data. These analyses often represent a collaborative effort between cytometry experts who generate the data and the quantitative individuals who help deal with the large volume information. In order for this collaboration to work, the cytometrists must have confidence in the results of the automated analysis. To this point, we demonstrate the equivalence of our results to those produced by an expert cytometrist using FlowJo.

## 2. Materials and Methods

### 2.1. Flow Cytometry Data

The data analyzed in this study was part of the initial set of experiments used to validate the BD FACS CAP platform. BD FACS CAP was designed as a cell characterization tool to screen for the presence of a large number of different human cell surface markers, and it was important to show that the assay was able to correctly identify positive and negatively staining markers on a well-studied cell population, such as PBMC lymphocytes. Previously frozen PBMC samples from two donors were analyzed on a BD FACS Calibur using BD FACS CAP staining plates. The analysis was performed on 96-well plates with 189 different antibodies arrayed three per well in 63 test wells, along with 30 isotype control wells and three unstained controls. The complete list of BD FACS CAP antibodies can be found at http://www.bd.com/technologies/discovery_platform/BD_FACS_CAP.asp. FCM files for the five plates (two for Donor 1 and three for Donor 2) are available for download from http://www.ficcs.org/data/plateData.tar.gz.

### 2.2. Data Analysis

**Figure 1:**
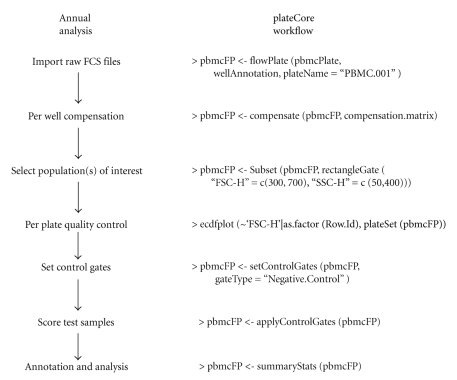
Typical FC-HCS plate workflow on the left and corresponding steps from a PBMC lymphocyte plateCore analysis on the right.

FCM output was analyzed in parallel using FlowJo and plateCore. Short descriptions of the steps in each software package are provided below. Additionally, the plateCore script used to perform the analysis is provided in Supplementary Materials available online at doi: 10.1155/2009/356141, and an example of the progression from raw FCM data files to a completed plateCore analysis for a single plate is shown in Figure 1.

### 2.3. plateCore

**(1) Template Construction**

A tab delimited text file was created that describes the contents of each well on the replicate plates. This information includes the marker name, fluorophore, antibody type, and the isotype group assignment. In this early version of BD FACS CAP the combination of antibodies in a well was based on available antibody-dye combinations. Newer versions of BD FACS CAP use biological information to assign markers to wells and are able extract more useful coexpression information.

**(2) Data Import**

FCM files for each plate were imported using flowCore. The import operation produces 5 flowSet objects, one for each plate, which were then integrated with the layout information in the template to create 5 flowPlates.

**(3) Gating**

flowPlates were processed using a combination of static gates (rectangleGate) and data driven gates (using norm2filter in flowCore) to pick out the lymphocytes in the forward (FSC) and side scatter (SSC) channels.

**(4) Plate Level Quality Assessment**

The quality of the data was then assessed by looking for fluidic events such as bubbles, pressure drops, or large aggregates that can shift the baseline fluorescence readings. Fluidic events can often be identified by plotting the empirical cumulative distribution function (ecdf) plots of FSC values for each well and looking for distributions shifted relative to other wells [9]. Based on the ecdf plots, several wells were further investigated by cytometry experts who determined that the shifts were in an acceptable range.

**(5) Isotype-Based Gating**

The threshold between positive and negative cells was determined using the isotype controls, which provided a gross estimate of nonspecific binding in the primary antibodies. One-dimensional gates were created using the isotype thresholds, and these gates were applied to identify cells that had specific staining in channels of interest. Details about the nonparametric isotype gating strategy implemented in plateCore are provided in the results section.

**(6) Summarization**

The 5 flowPlates were then aggregated into a single flowPlate using the fpbind operation from plateCore. Having the data in this format makes it easier to plot replicate wells from different plates, perform statistical analyses, and to export a single, experiment level results text file.

### 2.4. FlowJo

**(1) Template Construction**

An XML-based FlowJo template was created where test wells and their corresponding isotype control well were assigned to one of 30 groups. Wells in each group contained similar sets of antibody-dye conjugates.

**(2) Data Import**

FCM files were imported using the FlowJo template.

**(3) Gating**

Lymphocytes were selected using polygonal gates in the FSC-SSC view.

**(4) Plate Level Quality Assessment**

Quality assessment was performed by looking for wells where the FSC-SSC location of the lymphocyte population shifted relative to other wells on a plate.

**(5) Isotype-Based Gating**

Event data for isotype wells was visualized on a log scale, and the expression threshold for each stained channel was set by picking a value that lies above the bulk of the events. Isotype gates were initially set so that approximately 0.5% of the events in the isotype well were above the threshold. These gates were then applied to the test wells, and the gates were moved up or down depending upon positive and negative test well populations. If the population of cells in positive wells was much higher than the isotype gate, then the gate was moved up to help reduce false positives associated with nonspecific staining. Similarly, if the isotype gate was higher than negative samples, the gate would be moved down to ensure that positive cells were classified correctly.

**(6) Summarization**

The percentage of cells above the threshold for each of the 189 antibodies was then exported for each plate, and these results were merged to create the analysis report.

## 3. Results

Although this study focuses on comparing two different FC-HCS analysis methods, it is important to consider the original goal of the experiment used to generate the data when interpreting the results. BD FACS CAP was designed to provide a standard assay platform for screening a large number of markers on many different cell types. The validation effort for BD FACS CAP included running the assay on well-characterized cell types to find markers with either positive or negative staining and comparing these results to published cell expression profiles in literature. The PBMC lymphocyte staining results presented in the following section represent one of the cell types used for validating the technology.

### 3.1. FlowJo Output

Descriptions of marker expression profiles for particular cell populations in flow cytometry often use terms like positive-negative, or bright-dim, to qualify the amount of target present. Since BD FACS CAP is a standard platform for screening a wide range of cell types, and antibody concentrations were not optimized for these particular PMBC samples, results are reported as the percentage of cells above the isotype gate rather than positive or negative. Followup studies, including single color titrations and competition experiments, are needed to definitively show that a marker is present. Markers that have been previously characterized using BD FACS CAP with ≥90% of the cells above the isotype threshold are usually confirmed as positive using titration and competition experiments, while staining in markers with ≤10% of cells above the isotype threshold is often the result of nonspecific binding (data not shown). Note that these percentages refer to the fraction of cells above the isotype threshold, but this does not necessarily imply heterogeneous staining in multiple populations.

**Figure 2:**
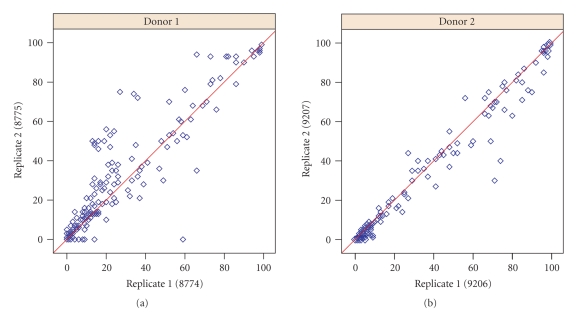
FlowJo estimates for the percentage of cells above the isotype threshold for 189 markers on replicate plates for donor 1 and donor 2. Estimates from markers where the center of the cell population was near the isotype threshold, around 50%, were more variable than samples which were clearly positive (≥99%) or negative (≤1%). The correlation for replicate plates was strong in both donors, with donor 1 at 0.92 and donor 2 at 0.98. Plate 9208 for donor 2 is not shown, since the results are very similar to 9206 and 9207.

Automating the creation and modification of isotype gates made by cytometrists analyzing BD FACS CAP data using FlowJo is challenging. Cytometrists adjust gates based on expert knowledge about the performance of specific antibody types and dyes, or after identifying positive or negative test samples. If the isotype gate cut off the bottom portion of a positive cell population in a test well, then the gate was moved down. Similarly, if the isotype gate included too many cells from negative test wells, it was moved up. Results from the FlowJo-based gating of replicate PBMC plates are shown in Figure 2. Detailed results for each marker are not presented in this study, but since the majority of antibodies on the BD FACS CAP staining plate are known to bind different leukocytes, it is not surprising that a large fraction would be identified as positive on PBMCs. Markers such as CD44, CD45, CD47, and CD59 are broadly expressed on lymphocytes and were positive (>99%) in this study.

### 3.2. plateCore versus FlowJo

Isotype controls are used to determine the threshold between background staining and specific binding of an antibody conjugate to its target. For the FlowJo analysis, the gate was initially set at the 99.5th quantile of the fluorescence signal in each stained channel of the isotype and then adjusted based on results from test wells. In plateCore, we have implemented two approaches to automatically creating gates based on negative controls. The first simply replicates the initial creation of the FlowJo gates and determines the threshold based on a set quantile, while the second uses a nonparametric approach where the gate (*G*_*ij*_) for isotype *i*, channel *j* was set according to

(1) $$G_{ij}={\text{MFI}}_{ij}+4{\text{MAD}}_{ij},$$

where MFI is the Median Fluorescence Intensity and MAD is Median Absolute Deviation in the raw data (linear scale). Although FCM fluorescence signals are approximately lognormal, as evident from density plots shown in this study (Figures 5  and 8), it is difficult to reliably make distributional assumptions, and the choice of 4 MADS represents a conservative attempt to set the gate above the 99th quantile of cells in the isotype stained wells.

The nonparametric gating approach is obviously more robust to outliers than a static gate based on the 99.5th quantile, but in practice both methods produce very similar results if the data is good quality and there are a sufficient number of cells (over 1000) in the isotype well. The plateCore analysis presented in this study used the nonparametric approach to gating, and while this relatively simple method works surprisingly well for BD FACS CAP, advances in model-based clustering methods, such as those in flowClust, should lead to future performance improvements in automated gating.

**Figure 3:**
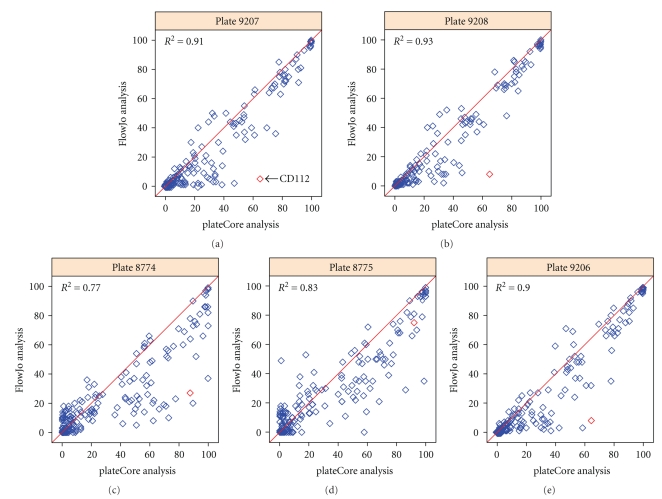
Plot showing the percentage of cells above the isotype threshold from plateCore (*x*-axis) and FlowJo (*y*-axis) for each of the 189 markers on the 5 PBMC plates. If the two methods produce similar estimates, then the values should be near the red line (*y* = *x*). In plateCore the isotype threshold was determined using only information from the isotype control well, while the threshold in FlowJo may be adjusted after identifying either positively or negatively staining test samples. Generally, these FlowJo adjustments resulted in the isotype gate being set a higher level to exclude a negative test sample. The effect of increasing the isotype threshold can be seen in these plots, where most disagreements are cases where plateCore estimates are higher than FlowJo. Detailed plots for one marker, CD112 (red diamond), where the two methods give different results are shown in Figure 5.

Comparisons of the output from the plateCore and FlowJo analyses are shown in Figure 3. Both methods produce nearly identical estimates for markers that were either clearly positive (≥99%) or clearly negative (≤1%), and R-squared values for all makers were between 0.83 and 0.93 (Figure 3). These cell populations are not close to the isotype threshold, and therefore different isotype gate settings have little or no effect on estimates of the percentage of cells above the gate. In situations where the isotype gate splits a test cell population, small changes to the gate can dramatically change these estimates. This effect is evident in the results from replicate plates using FlowJo (Figure 2) and in comparisons of FlowJo and plateCore (Figure 3), where estimates for markers having approximately 50% of the cells above the isotype gate are more variable than markers having ≤1% or ≥99%.

**Figure 4:**
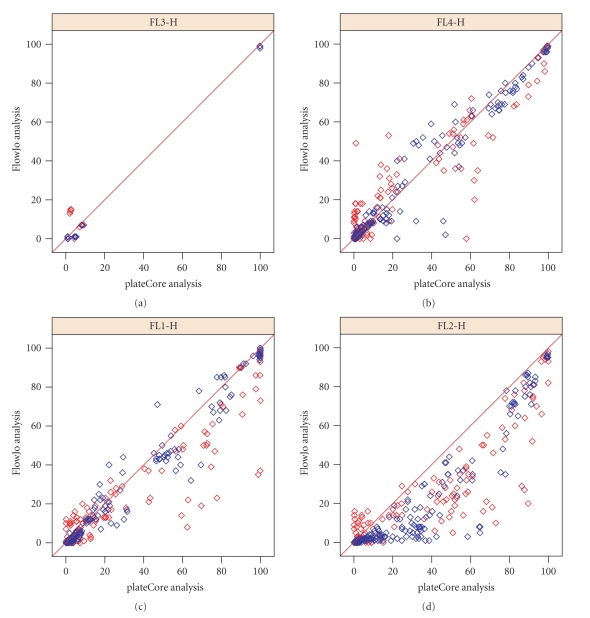
Plot showing the percentage of cells above the isotype threshold from plateCore (*x*-axis) and FlowJo (*y*-axis) for donor 1 (red) and 2 (blue) in channels FL1-H through FL4-H. plateCore gating for Phycoerythrin (PE) conjugated antibodies (FL2-H) was consistently lower than FlowJo, resulting in more cells above the isotype gate.

**Figure 5:**
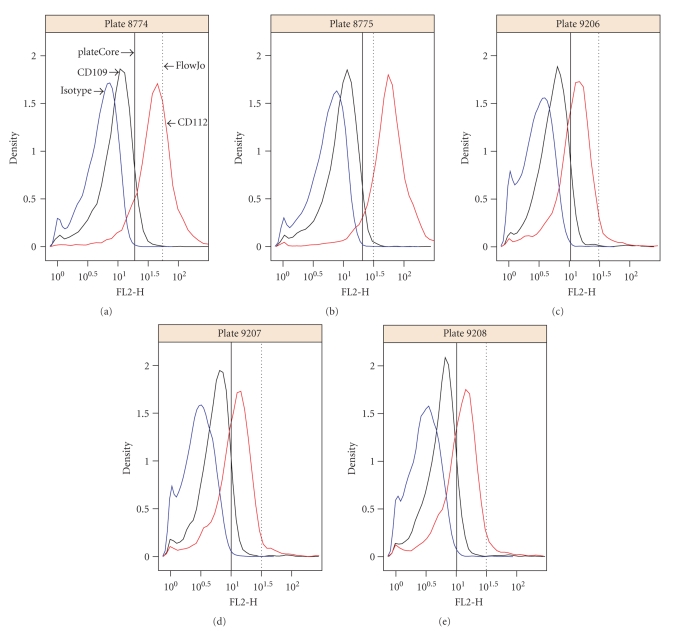
Density plots showing the plateCore (solid black) and FlowJo (dashed black) isotype gates for CD112 and CD109, which shared the same isotype control (IgG1-PE). The plateCore and FlowJo analyses gave different estimates for CD112 (see Figure 3), which was caused by the gate being moved higher in FlowJo based on the presumed negative staining for CD109.

Figure 4 shows the plateCore and FlowJo comparison broken down by channel, and we can see that a large portion of the markers that disagree were stained with Phycoerythrin (PE) in FL2-H. plateCore estimates for antibodies conjugated to PE were almost always higher than FlowJo, indicating that the isotype gates in FlowJo were moved above their initial setting. Looking in detail at one PE conjugate where the two methods disagree, CD112 IgG1-PE, we can see how the gate for was changed in the manual analysis based on what looks like nonspecific staining in a related test sample, CD109 IgG1-PE (Figure 5). Since the gene for CD112 (PVRL2) has been shown to be expressed on a subset of lymphocytes in healthy donors using microarrays [10], the plateCore results showing 65%–92% of the cells above the isotype gate may actually represent specific staining. Unfortunately, increasing the isotype (IGg1-PE) threshold in FlowJo to eliminate what looks like background staining in CD109 also seems reasonable. More focused studies will have to be performed to determine if the staining for CD112, and other markers that disagreed, was positive or negative.

### 3.3. Gating Quality Assessment

Since we may not always have access to output from expert cytometrists to help determine if our automated gating is reasonable, we need alternative approaches to assessing the quality of our isotype-based gates. The strategy we used for this PBMC study involves visually checking density plots of the isotype wells for replicate plates and also comparing the percentage of cells above the isotype gates versus the MFI ratio to see if the gating was consistent across the experiment. Plates for each PBMC donor are purely technical replicates; so any differences should be due to variation in cell staining or changes in instrument settings.

**Figure 6:**
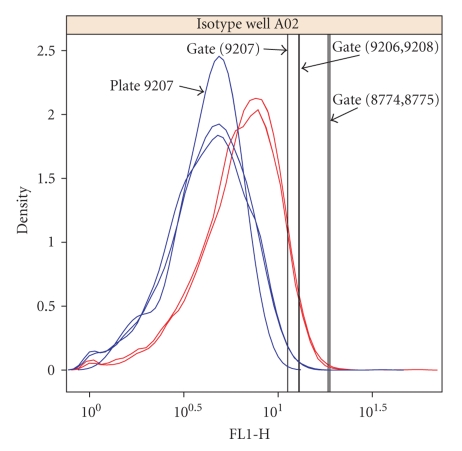
Density plot showing an example of one case where the isotype (IgG1-Alexa 488) gate settings differed between replicate plates for donor 2 (blue). In this case, the low setting for plate 9207 did not result in a significant difference between plates for the percentage of cells above the gate in the corresponding test well (CXCR5), so the gate was not modified. Plates 9206, 9207, and 9208 had 14%, 16%, and 15% percent of cells above the gate, respectively.

An example of the plots used to check replicate isotype gates is shown in Figure 6. In this case the threshold for one of the 3 replicate plates for donor 2 was lower than the other 2, indicating that the marker expression values from this isotype should be further evaluated. Fortunately, the difference is relatively small and did not change the estimate for the test well associated to this isotype (CXCR5 IgG1-Alexa 488). If the difference between replicates had been larger, we would have averaged the isotype thresholds from the remaining replicates and replaced the setting for plate 9207.

**Figure 7:**
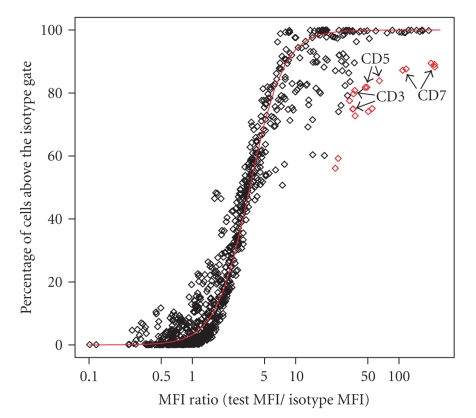
Quality of the automated gating was assessed by performing a robust logistic regression of the percentage of cells above the isotype gate on the log transformed MFI ratio and looking for estimates that were more than 2 standardized residuals away from the best fit line (red line). There were 18 estimates flagged in this study (red diamonds) where the value was different than we would predict from the MFI ratio. Detailed examination of these 18 cases showed that the isotype gate settings were reasonable, but they differed from other markers in that they had more than one population of stained cells. Sample density plots for one of these markers, CD3, are provided in Figure 8.

**Figure 8:**
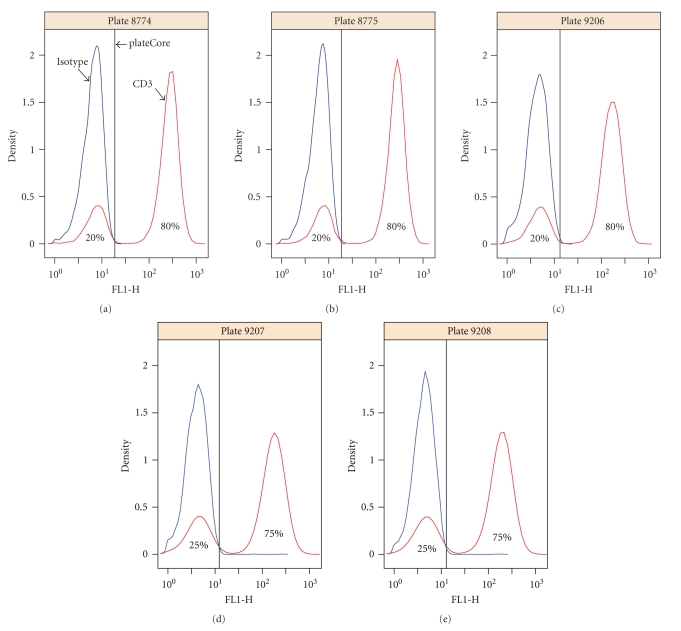
Density plot for CD3 (IgG1-Alexa 488), which was flagged for further evaluation by our gating quality assessment (Figure 7). The isotype gate settings look reasonable; however the MFI ratio for CD3 was very different from other markers that also had 75%–80% of their cells above the isotype gate. Looking at Figure 7, other markers with 75%–80% had MFI ratios near 5, while CD3 has an MFI ratio of 31–37. The flagging was the result of 2 cell populations for CD3, whereas most other markers stain a single population.

The MFI ratio is defined as the ratio of the MFI for a marker to the MFI of its isotype control. Essentially, this ratio tells us how well separated a population of stained test cells is from the population of cells in the isotype control. The distance between these two populations is related to the percentage of cells above the isotype gate (Figure 7). To evaluate isotype gating at the experiment level for these 5 plates we performed a robust logistic regression for the percentage of positive cells on the MFI ratio and looked for values that were more than 2 standard residuals from the best fit line. We chose 2 standard residuals in a conservative attempt to ensure that any questionable automated gating decisions were examined in detail. Deviation from the best fit line can indicate either a problem with the isotype gate or that the sample has multiple cell populations (Figure 8). If the percentage of cells above the gate is significantly different than we would predict from the MFI ratio, then the isotype gate was checked. We note that this approach does not actually tell us if the gating was correct, simply whether or not the isotype gating was consistent.

The bulk of the measured responses for the markers (927 out of 945) is within two standard residuals from the best fit line (Figure 7), which is surprising since the 189 different antibodies were conjugated to different fluorophores (either Alexa 488, FITC, PE, PerCP, APC, or Alexa 647) and matched against different isotypes (either IgG1, IgG2, IgG2a, IgG2b, IgG3, or IgM). We expected that differences in fluorescence intensity between dyes, and variation in nonspecific binding by different antibody types, would make direct comparisons difficult. The 18 values that were more than two standard deviations away from the line were examined in detail, and the isotype gate settings were found to be reasonable. In this case the flagging was the result of a positive and negative staining population of cells, which made the relationship between the MFI ratio and the fraction of cells above the isotype gate look very different than markers staining a single population. Density plots for one of the flagged markers, CD3, are shown in Figure 8.

## 4. Discussion

We were motivated to use the flowCore package for BD FACS CAP data analysis by a desire to reduce subjectivity associated with isotype gating and also to make the more analyses more reproducible. We found that while flowCore was very powerful, both in terms of efficient use of memory for large data sets and an extensive collection of FCM functions, it did not scale well to BD FACS CAP experiments with multiple plates and a complex layout. plateCore was developed to make it easier to perform operations and produce visualizations that are technically challenging to do in flowCore and flowViz. For example, creating a set of threshold gates based on negative control wells, either isotype or unstimulated cells, and then applying those gates to test wells on a plate is a relatively common FC-HCS operation. In this study, the PBMC isotype gates were created and applied to test wells in two steps, using setControlGates and applyControlGates (Figure 1). Replicating this same operation in flowCore would require either many individual custom gating steps or users to develop their own methods that duplicate the functionality in plateCore.

plateCore provided the ability to quickly analyze complex BD FACS CAP plates and produce useful visualizations (such as Figures 2–8), which facilitated discussions with the cytometry experts and helped to develop approaches to automate the gating process. Since this was a screening assay, the goal was to quickly and reproducibly process a large volume of data to get an approximate expression value for each of the 189 human cell surface markers and then perform more in-depth analysis for markers that were of biological interest. Using plateCore, we were able to reduce the level subjectivity in setting isotype gates, eliminate mistakes associated with manual data annotation and export, and automate the creation of plots and data quality reports that summarized the experiment. Additionally, the plateCore scripts and experimental annotation can be shared with other cytometry groups, allowing them to reproduce our analysis.

An important realization from our experience developing plateCore and analyzing BD FACS CAP experiments was that individual isotype gates should not be changed by cytometrists when performing FC-HCS experiments. The cytometrist does not have any information other than expert opinion about where a gate should go for a particular set of values, and making adjustments adds both bias and noise to the end result. In addition, the use of a more uniform gating approach facilitates the use of plateCore to combine and analyze results across many samples, which is one of the important new capabilities of this software. The functionality in plateCore enables cytometrists and statisticians to work together and make higher level decisions about gating strategies, based on methods like the gating quality assessment shown in Figure 7. Also, the gating in this experiment is relatively simple since we were only concerned with one dimension at a time. Developing new methods to reproducibly gate samples in three or more dimensions requires tools like flowCore and flowClust. plateCore provides infrastructure that makes the data available to quantitative scientists to further develop and apply these research tools.

The complexity of large FCM experiments, like BD FACS CAP, highlights the difficulty of applying existing FCM analysis platforms to high-throughput studies. Generating and interpreting results from this PBMC study required extensive collaboration between flow cytometrists, bioinformaticians, and statisticians. At various points in the analysis, each group needed to access the raw data, annotation, and details about the experimental design. Providing this access using stand-alone FCM platforms is expensive in terms of the price of multiple software licenses and in time spent training statisticians and bioinformaticians to use the programs. Fortunately the Bioconductor FCM packages are modeled on standard data structures used for microarrays, which should already be familiar to most quantitative individuals working on high-throughput biological problems. In addition, this approach allows scientists to use modern software development tools, including version control software, to manage plateCore scripts and make the analysis reproducible in a way that is generally not possible with GUI-based tools. Finally, we found that flowCore, flowViz, and plateCore provide an open analysis platform that facilitates communication between the flow cytometrists generating the data and the computational experts analyzing the data.

## Supplementary Material

Supplementary materials include:
1) the comma delimited percentagesRandFJ.csv file with the percentage of positive cells in each channel on each plate from both the plateCore and FlowJo analyses;
2) a detailed vignette (plateCoreVig.pdf) showing how to use the plateCore package and how to replicate the analysis.

## References

[1] Gasparetto M, Gentry T, Sebti S (2004). Identification of compounds that enhance the anti-lymphoma activity of rituximab using flow cytometric high-content screening. *Journal of Immunological Methods*.

[2] Brinkman RR, Gasparetto M, Lee S (2007). High-content flow cytometry and temporal data analysis for defining a cellular signature of graft-versus-host disease. *Biology of Blood and Marrow Transplantation*.

[3] Gentleman R, Carey VJ, Bates DM (2004). Bioconductor: open software development for computational biology and bioinformatics. *Genome Biology*.

[4] Maecker HT, Rinfret A, D'Souza P (2005). Standardization of cytokine flow cytometry assays. *BMC Immunology*.

[5] Hahne F, Le Meur N, Brinkman RR (2009). flowCore a bioconductor package for high throughput flow cytometry. *BMC Bioinformatics*.

[6] Sarkar D, Le Meur N, Gentleman R (2008). Using flowViz to visualize flow cytometry data. *Bioinformatics*.

[7] Gentleman R, Hahne F, Kettman J, Le Meur N Bioconductor package flowQ. http://www.bioconductor.org/.

[8] Lo K, Hahne F, Brinkman RR, Gottardo R (2009). flowClust: a bioconductor package for automated gating of flow cytometry data. *BMC Bioinformatics*.

[9] Le Meur N, Rossini A, Gasparetto M, Smith C, Brinkman RR, Gentleman R (2007). Data quality assessment of ungated flow cytometry data in high throughput experiments. *Cytometry A*.

[10] Critchley-Thorne RJ, Yan N, Nacu S, Weber J, Holmes SP, Lee PP (2007). Down-regulation of the interferon signaling pathway in T lymphocytes from patients with metastatic melanoma. *PLoS Medicine*.

