# Supplementary material for: Analysis of High-Throughput Flow Cytometry Data Using plateCore
Source: Adv Bioinformatics. 2009 Oct 11;2009:356141. doi: 10.1155/2009/356141 (PMC2777006; doi:10.1155/2009/356141)
Supplement: Supplementary file 1 — Supplementary materials include: 1) the comma delimited percentagesRandFJ.csv file with the percentage of positive cells in each channel on each plate from both the plateCore and FlowJo analyses; 2) a detailed vignette (plateCoreVig.pdf) showing how to use the plateCore package and how to replicate the analysis. [file 356141.f1.zip › plateCoreVig.pdf]

Analysis of High Throughput Flow Cytometry Data using *plateCore*

Errol Strain

July 20, 2009

Contents

1 Overview 1

2 Introduction 1
- 2.1 Background . . . 1
- 2.2 Bioconductor Flow Tools . . . 2

3 Installation and Getting Started 3

4 Creating a `flowPlate` 4

5 Compensation and Background Correction 7

6 Gating 9

7 Displaying Data 12

8 Extracting Results 14

9 Quality Assessment 16

10 Example: PBMC Large Panel Study 19

1 Overview

plateCore is a Bioconductor packaged created to make processing and analysis of large, complex flow datasets in R easier. High throughput flow studies are often run in a 96 or 384-well plate format, with a number of different samples, controls, and antibodies-dye conjugates present on the plate. Analyzing the output from the cytometer requires

keeping track of the contents of each well, matching sample wells with control wells, gating each well/channel separately, making the appropriate plots, and summarizing the results. *plateCore* extends the *flowCore* and *flowViz* packages to work on `flowPlate` objects that represent these large flow datasets. For those familiar with *flowCore* and *flowViz*, the gating (filtering), transformation, and other data manipulations for `flowPlates` are very similar to `flowSets`.

In this document we show how setup a *plateCore* analysis and provide examples for a publicly available dataset. The peripheral blood mononucleocyte (PBMC) dataset is a collection of five 96-well plates that have been stained with 189 different antibody-dye conjugates. The goal is to identify cells that are positively stained relative to some corresponding negative control.

2 Introduction

2.1 Background

plateCore was created to address the need for robust methods to analyze data from BD FACS™CAP experiments. The current version of FACS™CAP has 200+ antibody-dye conjugates on a single 96-well plate, where the antibodies are specific for different human cell surface markers or proteins. The output from an experiment is a per cell measurement of protein/marker expression for each antibody. FACS™CAP is designed to be a tool for screening a large number of surface markers on different types of human tissue or blood samples. Analyzing the data requires objective, high throughput approaches to gating and summarizing large amounts of flow data.

In addition to BD FACS™CAP, *plateCore* also works for any other type of plate-based flow data, or any collection of samples that can be organized into a `flowPlate` object. `flowPlates` are simply a convenient way to structure the data and manage the annotation for a set of related flow samples. Once the data is in *plateCore*, it's also easy to summarize results across multiple experiments and to integrate flow results with other types of data using Bioconductor and access statistical methods in R.

2.2 Bioconductor Flow Tools

plateCore is built on top of *flowCore* and *flowViz*, and the functionality implemented for `flowPlates` is a subset of what is available for `flowSets`. Getting your data into a `flowSet` is the best way to start exploratory analysis, and also gives you access to other flow packages such as *flowQ*, *flowStats*, and *flowClust*. Once the layout of the experiment has been standardized, loading the data into plateCore allows users to automate portions of the analysis, and makes summarizing the data and extracting the results easier.

3 Installation and Getting Started

Install the *plateCore* package and load the sample data containing a plate dataset, compensation files, and a table decsribing the layout of the experiment.

```
> library(plateCore)
> data(plateCore)
```

```
> ls()
```

```
[1] "compensationSet" "pbmcPlate"       "wellAnnotation"
```

The `pbmcPlate` is actually one of the 5 plates that make up the Peripheral Blood Mononucleocyte Cell (PMBC) dataset in the Section 10. This particular plate has 96 wells and is stained in the FL1.H (FITC), FL2.H (PE), FL3.H (PerCP.Cy5.5), and FL4.H (APC) channels. *flowCore* was used to read in the raw FCS files, and the `pbmcPlate` is a `flowSet` resulting from `read.flowset()` operation. `flowSets` are modeled on the microarray data structures used in Bioconductor. The `pbmcPlate` is comprised of an `exrs` expression matrix, along with the associated `phenoData`.

```
> pbmcPlate
```

```
A flowSet with 96 experiments.

  column names:
  FSC-H SSC-H FL1-H FL2-H FL3-H FL1-A FL4-H Time
```

The wellAnnotation `data.frame` describes the layout of the plate and the content of each well. Stained wells have a row for each of the channels of interest, while unstained wells only have a single row. The information in well annotation is similar to the `pData` matrix for `phenoData(AffyBatch)`. For `pData` all information about a sample is contained on a single row, which makes it difficult to handle a multiplexed flow dataset since each channel essentially requires multiple columns to describe the contents of a well. The wellAnnotation format is easier to create and maintain, and *plateCore* handles organizing the relevant information into a `pData` object and incorporating it into a `flowSet`.

```
> head(wellAnnotation)
```

```
  Well.Id Sample.Type Ab.Name Channel Negative.Control
1     A01     Isotype Isotype   FL1-H              A01
2     A01     Isotype Isotype   FL2-H              A01
3     A01     Isotype Isotype   FL4-H              A01
4     A02     Isotype Isotype   FL1-H              A02
5     A02     Isotype Isotype   FL2-H              A02
6     A02     Isotype Isotype   FL4-H              A02
```

The first column of the table contains a unique well identifier, which must correspond to one of of the samples from pbmcPlate. The FCS output from the cytometer usually contains the well id in the filename, and these filenames are what *flowCore* uses to assign sample names to a `flowSet`. These well identifiers must be unique to a well, which usually means using 3 character codes like "A01", "B09", "H10", etc. *plateCore* assumes that the first character provides the row name and that second two characters give the column on the plate. The second column in the annotation table gives the sample type, currently either "Unstained", "Test","Negative.Control", or "Isotype". Each well can only be a single sample type. The Ab.Name column contains either the name of the antibody, or some other descriptor that will be useful for making plots. Looking at well B05 in the FL4.H, we see that it is stained with an antibody named CDbd14.

```
> wellAnnotation[50,]

   Well.Id Sample.Type Ab.Name Channel Negative.Control
50     B05        Test  Cdbd14   FL4-H              A03
```

The antibody names in this PBMC dataset were masked prior to public release, so these names will not correspond to the standard CD names. The Channel column tells which channel was used to detect fluoresence for the antibody-dye conjugate. Negative.Control information indicates which well is the negative control (e.g. isotype) for test samples. The current version of plateCore only supports a single negative control well for each test well, but multiple test wells can use the same negative control. Each negative control well should be assigned as it's own negative control for reasons that will be explained in following sections.

Additional columns can be included in wellAnnotation and will be incorporated in later results, but the 5 columns described here are the minimum for a `flowPlate`.

4 Creating a `flowPlate`

Making a `flowPlate` requires a `flowset`, a well annotation table, and a name for the plate that is unique within the set of flow data under analysis. Later we may want to combine information from multiple plates, so having a unique plate name identifier (e.g. barcode) makes it easier to track the samples. Using the sample dataset from the *plateCore*, a `flowPlate` can be built.

```
> pbmcFP <- flowPlate(pbmcPlate,wellAnnotation,plateName="PBMC.001")
```

Looking at the first `pData` entry from the `pbmcPlate` and from `pbmcFP`, we can see how the `wellAnnotation` was incorporated into `pData`.

```
> pData(phenoData(pbmcPlate))[1,]

[1] "000001_0877408774.A01"
```

```
> pData(phenoData(pbmcFP))[1,]
```

```
                                  name Well.Id plateName   FL1.H
0877408774.A01 000001_0877408774.A01     A01  PBMC.001 Isotype
               Sample.Type.FL1.H Negative.Control.FL1.H   FL2.H
0877408774.A01           Isotype                    A01 Isotype
               Sample.Type.FL2.H Negative.Control.FL2.H   FL4.H
0877408774.A01           Isotype                    A01 Isotype
               Sample.Type.FL4.H Negative.Control.FL4.H FL3.H Sample.Type.FL3.H
0877408774.A01           Isotype                    A01  <NA>              <NA>
               Negative.Control.FL3.H Sample.Type Row.Id Column.Id
0877408774.A01                   <NA>        <NA>      A        01
```

The following examples use the lymphocyte population from `pbmcFP`, so we select the cells of interest using a `rectangleGate` and `norm2Filter`. Details on how to use `Subset()` can be found in the Gating section and in *flowCore*. The cells selected by this gate are shown in Figure 1.

```
> rectGate <- rectangleGate("FSC-H"=c(300,700),"SSC-H"=c(50,400))
> normGate <- norm2Filter("SSC-H","FSC-H",scale.factor=2.5)
> pbmcFP.lymph <- Subset(pbmcFP, rectGate & normGate)
```

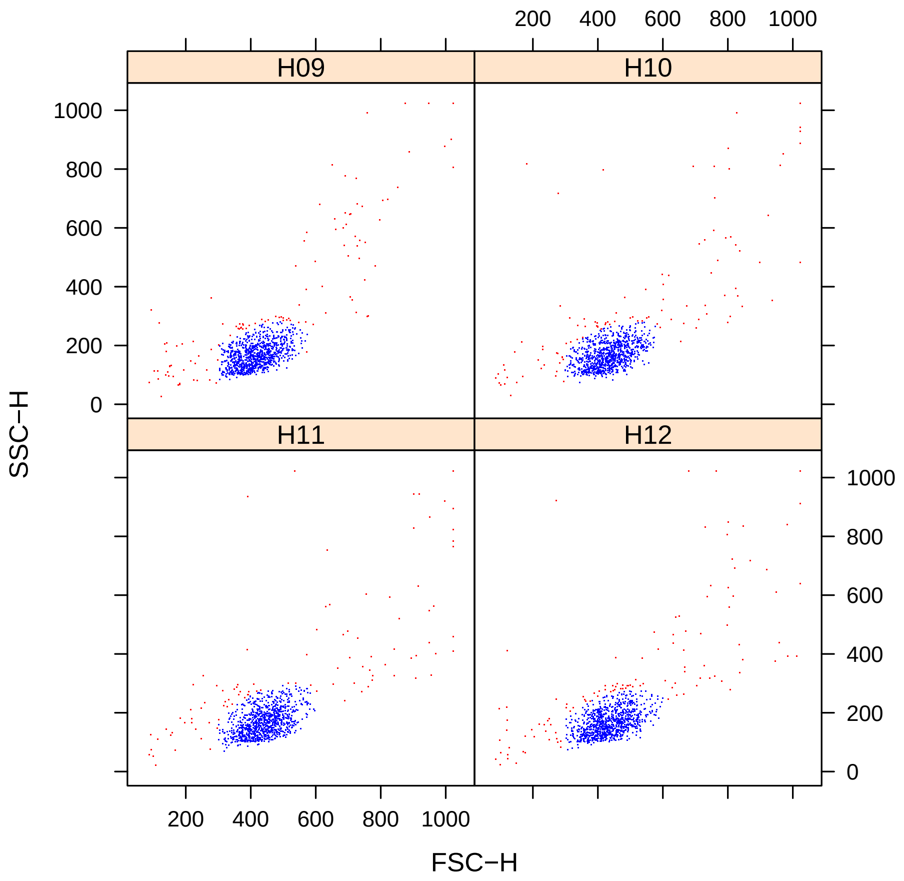

Figure 1: Forward (FSC) and side-scatter (SSC) dotplots for the first four wells of the PMBC plate. The lymphocyte population is shown in blue while monocytes are located in the upper right corner. Lymphocytes were selected using the `rectangleGate` and `norm2Filter` gates from *flowCore*.

5 Compensation and Background Correction

The `compensation()` function from *flowCore* is used to create a compensation matrix for the sample data. Channel names in the compensation `flowSet` must match the dataset under analysis, otherwise the matrix will not work correctly. Details about how `compensation()` works can be found in *flowCore*. We can create a compensation matrix using the sample data in *plateCore* and the `spillover()` from *flowCore*.

```
> comp.mat <- spillover(x=compensationSet,unstain=sampleNames(compensationSet)[5],
+ patt=".*H",fsc="FSC-H",ssc="SSC-H",method="median")
```

This `comp.mat` matrix can then be applied to a `flowPate` to correct for the effects of spillover. The difference between compensating in *flowCore* and compensating in *plateCore* is that *plateCore* only compensates for the dyes listed in `wellAnnotation`, whereas *flowCore* compensates each sample the same way. Since *plateCore* does this custom compensation, it is important to list each dye or fluorophore in the wellAnnotation, even if the sample will not be used for further analysis. If all the samples are stained the same way, then compensating with either approach should give the same results. The `pbmcFP` can be compensated using the `comp.mat` from above.

```
> pbmcFPcomp <- compensate(pbmcFP.lymph,comp.mat)
```

In this particular case the `pbmcPlate` has already compensated on the cytometer, so no further compensation is necessary.

The process of analyzing and gating (filtering) the large amount of data in a `flowPlate` can be simplified by first correcting for the effects of cell size on background fluoresence. This correction will later allows us to define a one-dimensional gate between positive and negative cells in only the channel interest, instead of having a two dimensional gate that includes the forward scatter (FSC) channel. The background correction uses the unstained wells and fits a log-log linear model to FSC versus each fluorescence channel (Insert Florian's ref). The correction is then applied to all the wells on a the plate. The `fixAutoFl()` function takes a `flowPlate`, and character variables indicating the FSC channel and the fluorsence channels (chanCols).

```
> pbmcFPbgc <- fixAutoFl(pbmcFP.lymph,fsc="FSC-H",chanCols=rownames(comp.mat))
```

Dotplots for before and after the background correction are shown in Figure 2. The result of the background correction on lymphocytes is subtle at best, but it can have dramatic effects for other cells types that have a wider range of FSC values.

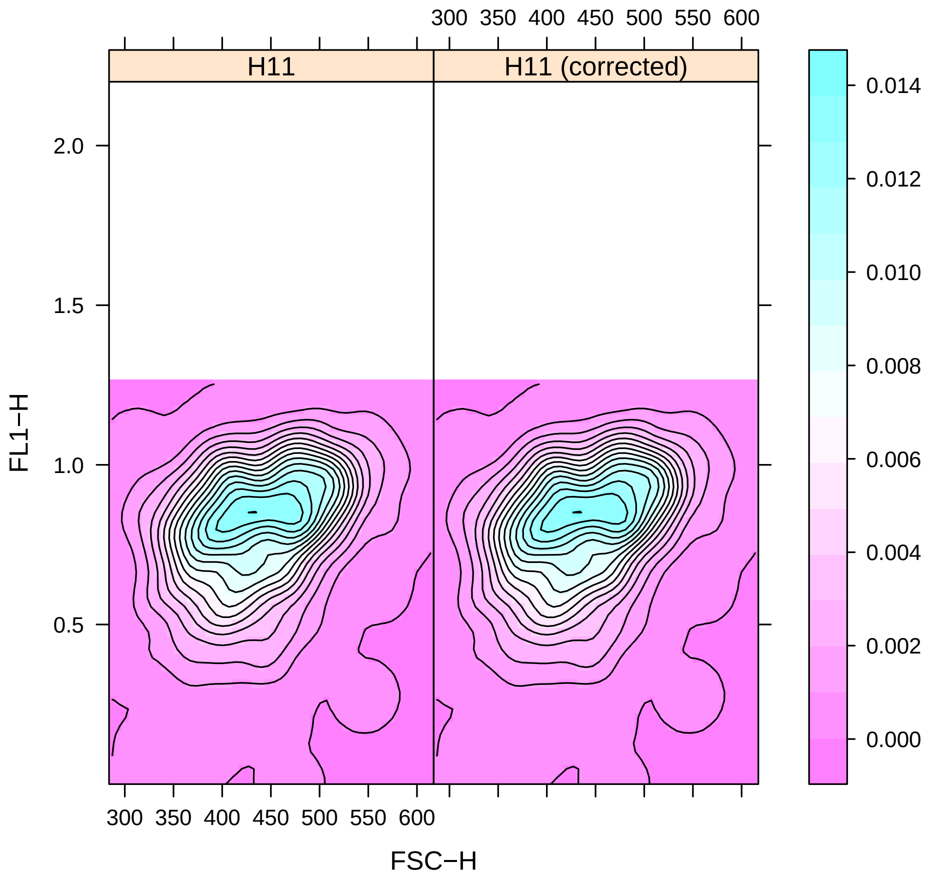

Figure 2: Dotplots showing the effects of cell size (FSC) on fluorescence for the unstained cells before (left) and after (right) the background correction. In the uncorrected data the fluorescence signal generally increases with increasing cell size (FSC).

6 Gating

Gating on `flowplates` is mainly performed using `Subset()`. The first argument to this function is a `flowPlate` and the second is a valid *flowCore* filter(s). For exploratory analysis and setting up the initial gates, it is more convenient to work with a `flowSet`, which can be extracted from a `flowPlate` using the `plateSet()` function.

```
> fs <- plateSet(pbmcFP)
```

For example, we can use a `rectangleGate` to separate the lymphocytes from the debris and monocytes. Looking at the plots in Figure 1, the lymphocytes look like they are located from about 400 to 650 on the FSC scale, and 100 to 300 on the SSC scale. We can use `xyplot()` from the *flowViz* package to display the gates.

```
> rectGate <- rectangleGate("FSC-H"=c(400,700),"SSC-H"=c(100,300))
> xyplot(`SSC-H` ~ `FSC-H` | as.factor(Well.Id), fs[1:2], displayFilter=TRUE,
+ smooth=FALSE, col=c("red","blue"),filter=rectGate)
```

Since we want this gate to be applicable to all the wells, it may help to enlarge the `rectangleGate` and then use a data-driven gate like `norm2Filter` to pick out lymphocytes. The lymphocyte population may drift over the course of analyzing the plate. `norm2Filter` will fit a bivariate normal to the FSC and SSC channels to identify an elliptical region of high density.

```
> rectGate <- rectangleGate("FSC-H"=c(300,700),"SSC-H"=c(50,400))
> normGate <- norm2Filter("SSC-H","FSC-H",scale.factor=1.5)
> xyplot(`SSC-H` ~ `FSC-H` | as.factor(Well.Id), fs[1:4], displayFilter=TRUE,
+ smooth=FALSE, col=c("red","blue"),filter=rectGate & normGate)
```

The results of applying the `rectangleGate` and `norm2filt` are shown in Figure 1.

Once the population of interest has been identified, `Subset()` the `flowPlate` to select those cells. These same gates were used to select lymphocytes when we were initially creating the flowSet. If your sample is comprised of multiple cell populations, as in this PBMC example, then it is necessary to `Subset()` before the compensation and background correction steps. The `compensationSet` should be processed with the `rectangleGate` and `norm2Filter` gates prior to constructing the compensation matrix.

```
> rectGate <- rectangleGate("FSC-H"=c(300,700),"SSC-H"=c(50,400))
> normGate <- norm2Filter("SSC-H","FSC-H",scale.factor=1.5)
> pbmcFP.lymph <- Subset(pbmcFP, rectGate & normGate)
```

Now we're ready to set the isotype (Negative.Control) gates that will be used to idenfity positively and negatively stained cells. We will estimate the gates using the `flowPlate` that has been subsetted for lymphocytes, compensate, and then background corrected. The default settings for the `setControlGates()` assumes the data is on a linear scale.

```
> pbmcFPbgc <- setControlGates(pbmcFPbgc,gateType="Negative.Control",numMads=5)
> pbmcFPbgc <- applyControlGates(pbmcFPbgc)
```

The `numMads` parameter indicates how far above the isotype population the positive-negative threshold is set. The threshold is set by taking the median fluorescence intensity (MFI) and adding 5 median absolute deviations (MADs). The default value of 5 works well for a number of cell types but may need to be adjusted for specific applications. Future versions of *plateCore* will use more sophisticated methods of estimating kernel density to fit distrubutions to the data and have a more robust esimtate of the positive-negative threshold. Once the control gates have be created, they are applied to the test wells using the `applyControlGates()`.

We see how reasonable the gates look using `xyplot()`. Examples of the test wells associated with the negative control well A03 are shown in Figure 3. To check all the gates in the FL1.H channel we would use the following code.

```
> xyplot(`FL1-H` ~ `FSC-H` | as.factor(Well.Id),
+ transform("FL1-H"=log10) %on% pbmcFPbgc, displayFilter=TRUE,
+ smooth=FALSE,col=c("red","blue"),filter="Negative.Control")
```

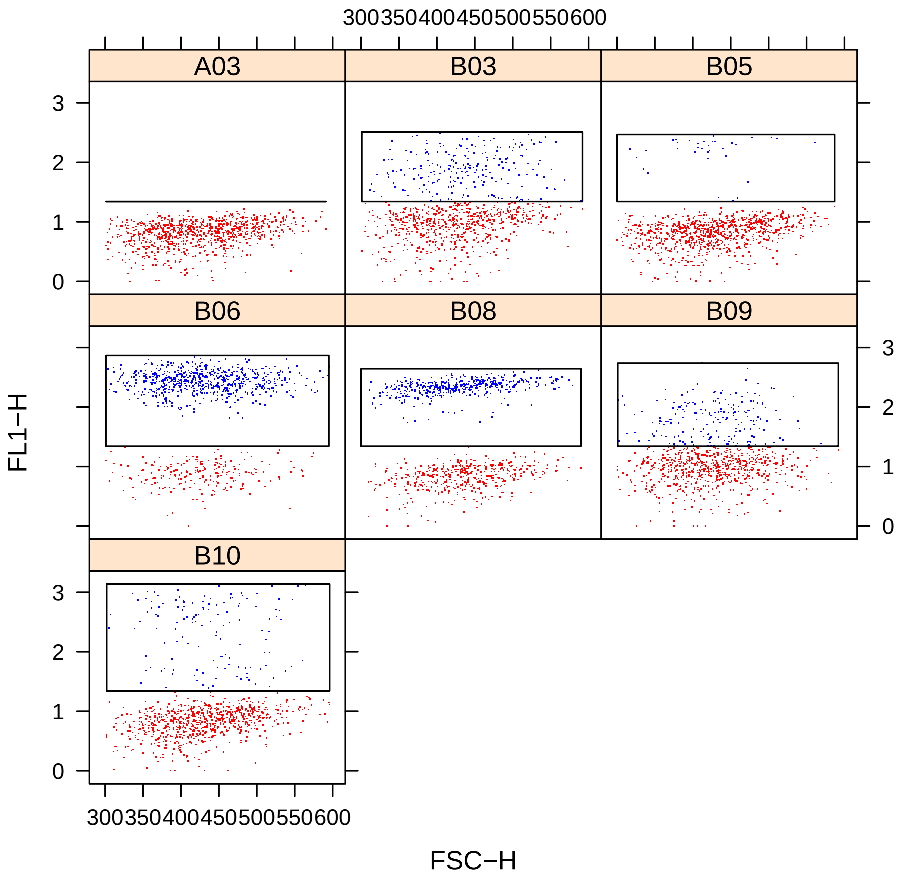

Figure 3: Dotplots showing the test wells associated with the negative control well A03. The gate was created automatically using `setControlGates()` to estimate the threshold between positive and negative cells based on the staining in A03. `rectangleGates` are drawn around the positive cells in each plot. Positive cells are shown in blue and negative cells in red.

7 Displaying Data

Tools for visualizing `flowPlates` are based on the collection of functions in *flowViz*, which is itself based on *lattice*. Currently on `xyplot()` works directly on a `flowPlate` object, but the other functions like `levelplot()` (shown in Figure 2) can be used by accessing the `flowSet` inside a `flowPlate` via `plateSet()`. `xyplot()` is the primary tool for making dotplots, showing gates, and creating overlay plots.

If the negative control gates have been created using `setControlGates()`, then they can be shown using `xyplot()`. The code used to generate Figure 3 is shown below.

```
> wells <- unique(subset(pbmcFPbgc@wellAnnotation,Negative.Control=="A03",
+   select="name")[,1])
> xyplot(`FL1-H` ~ `FSC-H` | as.factor(Well.Id),
+   transform("FL1-H"=log10) %on% pbmcFPbgc[wells],
+   displayFilter=TRUE,smooth=FALSE, col=c("red","blue"),
+   filter="Negative.Control")
```

The wells of interest are identified by looking for all the wells that have A03 are their negative control. When used for plotting `flowSets`, the filter argument takes a *flowCore* `filter`. For `flowPlates` the filter can also accept a character string indicating what type of gate to display. *plateCore* currently supports "Negative.Control" gates, and more options will be available in the future.

In the plots shown in Figure 3, a number of the test samples are heterogenous in expression for the markers in the FL1.H channel. For these samples it is clear that the gate created using `setControlGates()` looks reasonable. In other situations where the difference between positive and negative cells is not as distinct, making overlay dotplots showing both the test and negative control samples on the same graph can help to determine the appropriate gate. An example of the code use to generate the overlay plots in Figure 4is shown below.

```
> wells <- unique(subset(pbmcFPbgc@wellAnnotation,Negative.Control=="A06",
+   select="name")[,1])
> xyplot(`FL1-H` ~ `FSC-H` | as.factor(Well.Id),
+   transform("FL1-H"=log10) %on% pbmcFPbgc[wells],
+   displayFilter=TRUE,smooth=FALSE, col=c("blue","green"),
+   filter="Negative.Control",filterResults="Negative.Control")
```

In order for the gates to display correctly, any transformations must be applied directly on the `flowPlate`. This ensures that the Negative.Control gates, along with the fluorescence signal data, are on the same scale. If the transformation is in the formula, as in the following example, the the gates will not show up.

```
> xyplot(log10(`FL1-H`) ~ `FSC-H` | as.factor(Well.Id), pbmcFPbgc[wells],
+ displayFilter=TRUE,smooth=FALSE, col=c("blue","green"),
+ filter="Negative.Control",filterResults="Negative.Control")
```

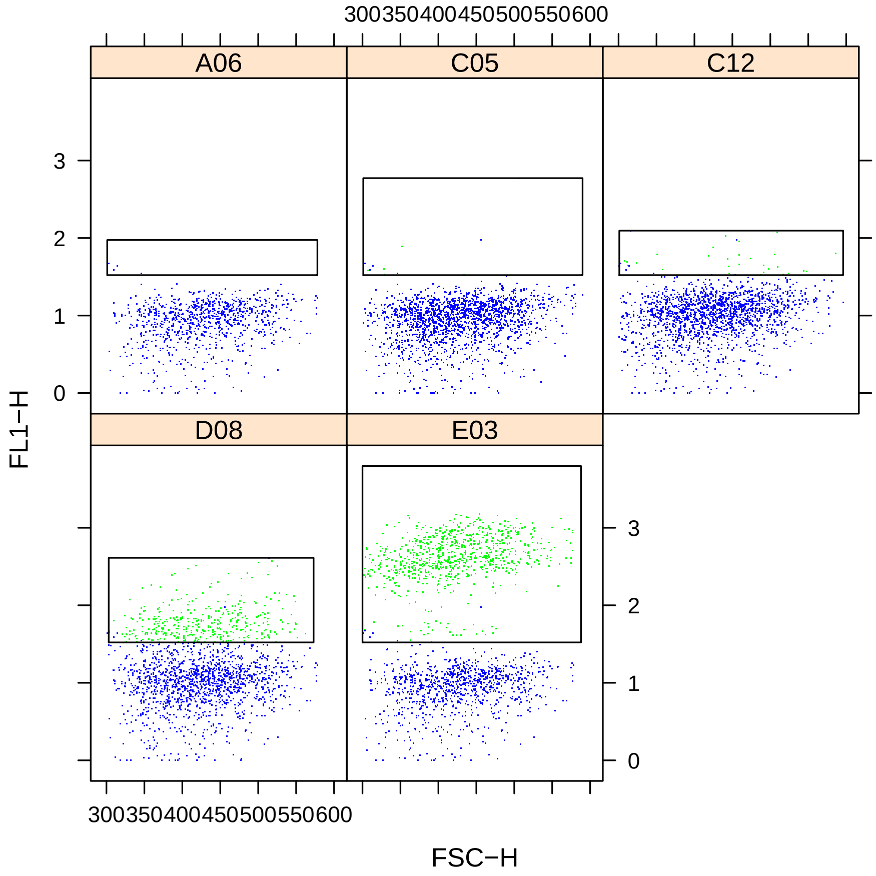

Figure 4: Overlay dotplots for the test wells associated with the negative control well A06. The cells from A06 (green) are shown in each dotplot along with the results from the test cells (blue). In cases where the test well population is close to the positive-negative threshold, such as well D08, these plots can help determine if the Negative.Control gates should be adjusted.

8 Extracting Results

Once the Negative.Control gates have been created and applied, we can then use the `summaryStats()` to calculate different metrics of interest from the `flowPlate`. Running `summaryStats()` on the `pbmcFPbgc`,

```
> pbmcFPbgc <- summaryStats(pbmcFPbgc)
```

will result in additional columns created in the associated `wellAnnotation` `data.frame`.

```
> colnames(pbmcFPbgc@wellAnnotation)

 [1] "Well.Id"               "Sample.Type"           "Ab.Name"
 [4] "Channel"               "Negative.Control"      "plateName"
 [7] "name"                  "Negative.Control.Gate" "Percent.Positive"
[10] "Total.Count"           "Positive.Count"        "MFI"
[13] "MFI.Ratio"             "Predict.PP"            "Gate.Score"
```

These new columns include percentage of cells above the Negative.Control gate (Percent.Positive), the number of cells in the raw data (Total.Count), the number of positive cells (Positive Count), the median fluorescence intensity (MFI), and the ratio of the test well MFI to the MFI of the negative control well (MFI.Ratio). The Predict.PP and Gate.Score columns are explained in Section 9. In this PMBC example a number of the samples have multiple cell populations, so the MFI and MFI.Ratio may not be helpful since they are based on all the cells in a well, and not just the positive cells.

Now that we have this information we can display it using `xyplot()`. We can add the marker names and percent positive results to our dotplots (Figure 5).

```
> wells <- unique(subset(pbmcFPbgc@wellAnnotation,Negative.Control=="A03",
+          select="name")[,1])
> print(xyplot(`FL1-H` ~ `FSC-H` | as.factor(Well.Id),
+   transform("FL1-H"=log10) %on% pbmcFPbgc[wells],
+   displayFilter=TRUE,smooth=FALSE, col=c("blue","green"),
+   filter="Negative.Control",
+   flowStrip=c("Well.Id","Ab.Name","Percent.Positive")))
```

The `wellAnnotation` `data.frame` can be exported as a comma delimited for a record of the analysis.

```
> write.csv(pbmcFPbgc@wellAnnotation,file="PMBC.001.csv")
```

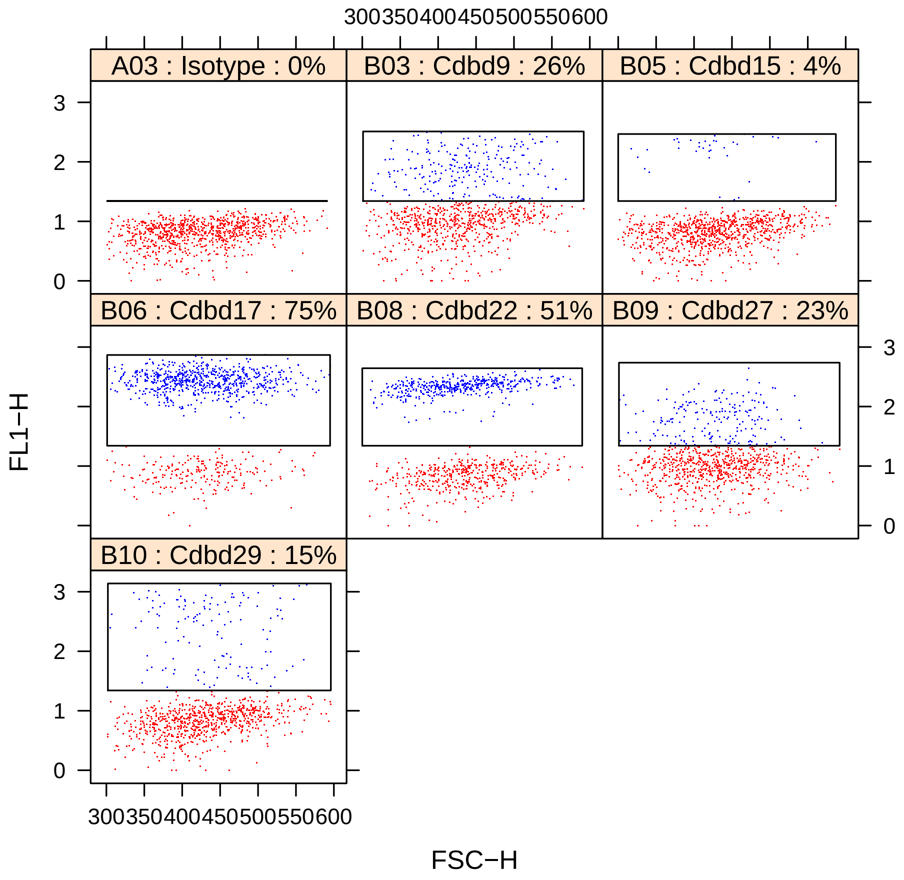

Figure 5: Dotplots and Negative.Control gates for wells associated with control well A03. Negative cells are shown in read and positive cells in blue. The strip above each plot now contains the Antibody-Marker name, along with the percentage of positive cells in the well.

9 Quality Assessment

Quality assessment using Bioconductor flow tools is covered in detail in the flowQ publication (Le Meur et al. 2007, Cytometry Part A). This example briefly covers how to check the number of lymphocyte events in each well, scan for fluidic events, and check the consistency of the isotype gating.

The number of gated lymphocytes in each well of the pbmcFPbgc dataset can be found in the Total.Count column of the wellAnnotation.

```
> summary(wellAnnotation(pbmcFPbgc)$Total.Count)

   Min. 1st Qu.  Median    Mean 3rd Qu.    Max.    NA's 
  858.0   885.0   890.0   888.9   894.0   903.0     3.0 
```

Since each well has at least 858 gated lymphocyte events there are no apparent problems with the sample acquisition on the plate.

Fluidic events (bubbles, etc.) can cause the cytometer readings to shift. These type of events can often be identified by plotting FSC vs Time, or through ecdf plots (as described in Le Meur et al. 2007, Cytometry Part A). An example of an ecdf plot for FSC looking for a row effect, since the samples are acquired by row, is shown in Figure 6. The code used to produce the figure is shown below.

```
> print(flowViz::ecdfplot(~`FSC-H` | as.factor(Column.Id),
+  data=plateSet(pbmcFPbgc), groups=Row.Id, auto.key=TRUE))
```

One approach to evaluating the consistency of isotype-based gating is to plot the MFI ratio versus the percentage of positive cells. The MFI ratio is the ratio of the median fluorescence intensity of the test sample to its corresponding isotype control. If two sample have approximately the same percentage of positive cells, then their MFI ratios should be similar. The `summaryStats()` function in *plateCore* performs a robust logistic regression on the MFI ratio to the percentage of positive cells, and results from the fit are stored in the Predict.PP and Gate.Score columns of wellAnnotation. Predict.PP gives the estimated percentage of positive cells based on the MFI ratio, and Gate.Score indicates how many standard residuals a sample data point is from the best fit line. Figure 7 shows the MFI ratio versus percent positive plot for the PBMC lymphocyte example. The code used to generate the plot is shown below. The `glmrob()` function from the *robustbase* package is used to perform the regression.

```
> mfiPlot(fp,thresh=2,xlab="MFI Ratio (Test MFI / Isotype MFI)",xlim=c(0.1,250),
+                 ylab="Percentage of cells above the isotype gate",pch=23)
```

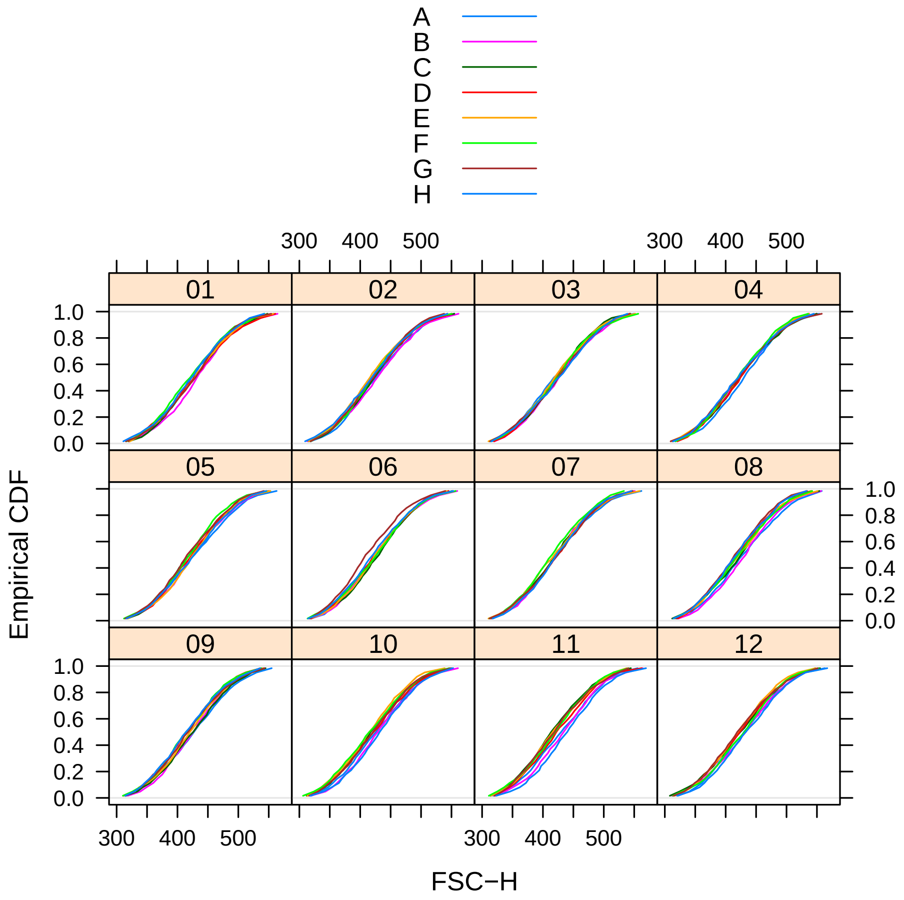

Figure 6: Empirical Cumulative Distribution Function (ecdf) plot for FSC from the pmbc lymphocyte example plate.

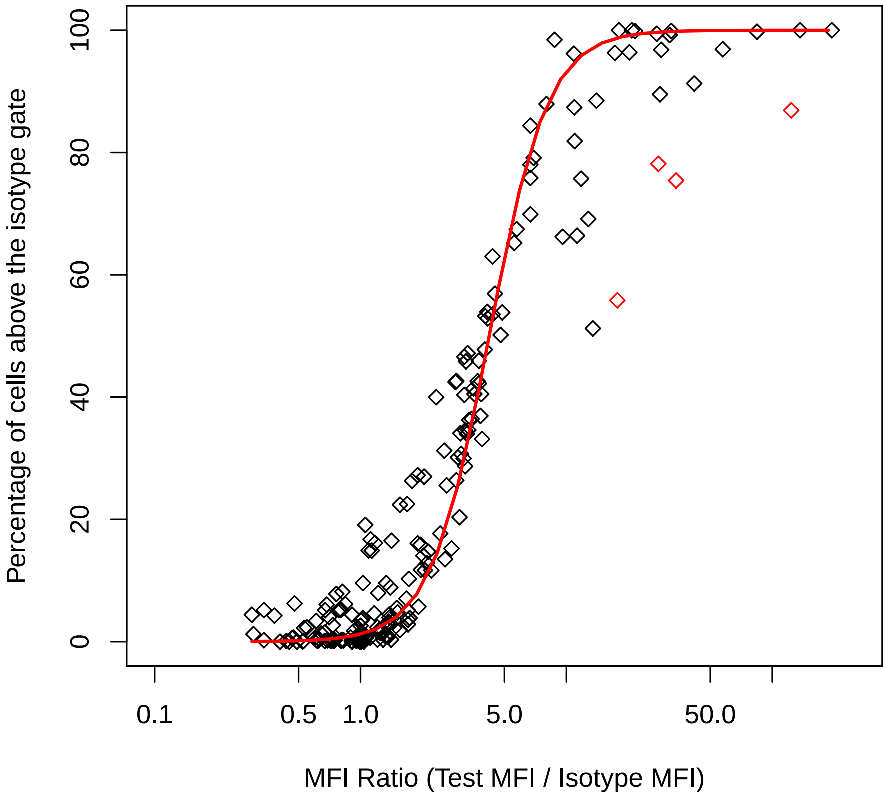

Figure 7: Plot of the MFI ratio versus the percentage of positive cells for the example PBMC lymphocytes. The robust best fit line is shown in red, and samples that are more than 2 standard residuals away from the line are shown in red.

10 Example: PBMC Large Panel Study

The PBMC dataset used in this example is available for download from ficcs.org as the plateData.tar.gz file. The data consists of 5 different PBMC samples that were analyzed with 189 different antibodies on 96-well plates. Each plate has a set of unstained, isotype, and control wells. Antibodies and isotype controls are arrayed 3 per well, but the data was compensated on the cytometer so there is no need to correct for spillover. This example assumes that the plates have been unpacked and stored in the folder "data/pbmc". Each of the 5 subfolders in the PBMC directory contains 96 fcs files. The text delimited file describing the layout of the plate is named pmbcPlateLayout.csv.

Processing all 5 plates is memory intensive, so each plate was loaded separately and the resulting `flowPlate` was stored in an R data image in the pbmcRData folder. The code used to process plate 8774 is shown below. Settings for the remaining 4 plates were identical to plate 8774.

```
> plateName <- "lymph08774"
> plateDescription <- read.delim("pmbcPlateLayout.csv",
+          as.is=TRUE,header=TRUE,stringsAsFactors=FALSE)
> platePBMCraw <- flowPlate(data=read.flowSet(path="data/pbmc/08774"),
+          plateDescription,plateName=plateName)
> platePBMC <- Subset(platePBMCraw,
+          rectangleGate("FSC-H"=c(300,700),"SSC-H"=c(50,400)) &
+          norm2Filter("SSC-H","FSC-H",scale.factor=1.5))
> platePBMC <- setControlGates(platePBMC,gateType="Negative.Control")
> platePBMC <- applyControlGates(platePBMC)
> platePBMC <- summaryStats(platePBMC)
> save.image(file=paste("pbmcRData/",plateName,".Rdata",sep=""))
```

Once the 5 plates have been processed, the `flowPlates` can then be combined using `fpbind()`. In this case the plates were saved using the same name, platePBMC. The `flowPlates` were read into a list, and then combined into a large "virtPlate".

```
> fileNames <- list.files("pbmcRData",full.names=TRUE)
> plates <- lapply(fileNames,function(x){
+                           load(x)
+                           platePBMC
+                  })
> virtPlate <- fpbind(plates[[1]],plates[[2]],plates[[3]],plates[[4]],plates[[5]])
```

Once the plates have been combined, xyplots and densityplots can then be conditioned on "plateName" to create graphics like Figure 8. The R code used to generate Figure 8 is shown below.

```
> print(densityplot(~ `FL2-H` | as.factor(plateName),
+          transform("FL2-H"=log10) %on% virtPlate[c("C02","C03","A05")],
```

```
+           layout=c(3,2),xlim=c(-0.2,2.5),
+           filterResult="Negative.Control",lty=c(1,2,3,4),
+           col=c('blue','black','red')))
```

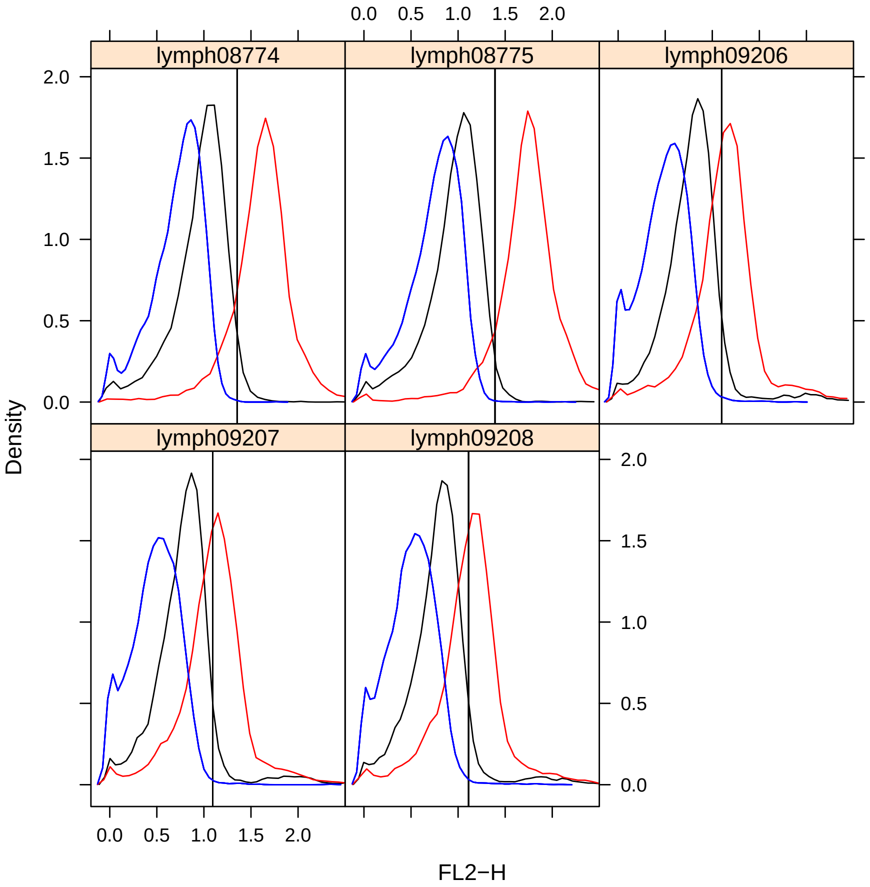

Figure 8: Density plot for two test antibodies (red,black) and their associated isotype control (blue). The isotype gate is indicated with a vertical black bar.
